# Glutathione S-transferase genes variants and glioma risk: A case-control and meta-analysis study

**DOI:** 10.7150/jca.29398

**Published:** 2019-08-19

**Authors:** Weiping Liu, Hongyu Long, Mengqi Zhang, Yanjing Wang, Qiong Lu, Haiyan Yuan, Qiang Qu, Jian Qu

**Affiliations:** 1 Department of Neurology, Xiangya Hospital, Central South University, Changsha 410008, People's Republic of China; 2 Department of Neurosurgery, Xiangya Hospital, Central South University, Changsha 410008, People's Republic of China; 3 Department of Pharmacy, the Second Xiangya Hospital, Central South University; Institute of Clinical Pharmacy, Central South University, Changsha 410011, People's Republic of China.; 4 Department of Pharmacy, Xiangya Hospital, Central South University, Changsha 410078, People's Republic of China

**Keywords:** glutathione S-transferase genes, glioma, gene polymorphism, risk, meta-analysis

## Abstract

**Background:** The glutathione S-transferase (GST) genes encode enzymes that metabolize carcinogenic compounds, and their variants, *GSTP1* (Ile105Val and Ala114Val), *GSTT1* (null/present)*,* and *GSTM1* (null/present)*,* reduce enzyme activity that may affect the risk of developing cerebral glioma. This study undertook a case-control study and a meta-analysis to evaluate associations between these GST gene variants and the risk of glioma.

**Methods:** The study enrolled 384 glioma patients (194 men and 190 women; mean age, 48.3 ± 9.2 years) and 340 healthy controls (174 men and 166 women; mean age, 46.5 ± 9.8 years). The amplification refractory mutation system assay was performed to identify GST gene variants of all 724 subjects. A meta-analysis enrolled 15 studies (including our case-control results) was performed.

**Results:** Our case-control study found that the frequency of *GSTP1* Ile105Val Val/Val genotype was significantly higher in the glioma group than that in the healthy controls (11.7% *vs.* 6.4%) (OR=1.50; 95% CI=1.05-2.04; P=0.01); the frequency of the Val/Ile + Ile/Ile genotypes was different from glioma patients and controls (88.3% *vs.* 93.6%) (OR=1.47(1.04-2.10); P=0.015); there were no associations between *GSTP1* Ala114Val,* GSTT1* (null/present) and *GSTM1* (null/present) variants and glioma risk. Our meta-analysis confirmed that the *GSTP1* Ile105Val variant was associated with an overall increased glioma risk. Moreover, our meta-analysis also confirmed the *GSTP1* Ala114Val and *GSTT1* null/present variants were associated with an increased glioma risk in the Caucasian population, rather than the Asian population.

**Conclusions:** This study showed that GST gene variants were associated with an increased risk of glioma with ethnic differences. Future large-scale, multi center, controlled, prospective studies are required to support these findings and to determine how these GST gene variants may affect the pathogenesis of glioma.

## Introduction

Glioma accounts for approximately 80% of all brain tumors and are associated with poor patient survival 1. To the best of our knowledge, no environmental carcinogens, apart from ionizing radiation, have been implicated in the etiology of gliomas 2-4. However, studies in rats demonstrated that brain tumors maybe induced by various carcinogenic substances, such as acrylonitrile, ethylene oxide and acrylamide 5-7. However, studies in humans reported no definitive association with occupational or environmental exposure and the risk of primary brain tumor 8-10. Several individuals may be exposed to toxic agents over time. Therefore, some researchers have focused on the polymorphisms in the glutathione-S-transferase (GST) A (*GSTM1*), u (*GSTT1*) and k (*GSTP1*) variants, which have been reported to be associated with the etiology of cerebral tumors 11-23. These enzymes, as part of the phase II detoxification process, are involved in the metabolism of several electrophilic compounds, including carcinogens, mutagens, cytotoxic drugs and metabolites, and the detoxification of the products of reactive oxidation 24-26.

Previously published studies have shown that variants of the *GSTP1* gene (Ile105Val and Ala114Val), variants of the *GSTT1* gene (null/present), and variants of the *GSTM1* gene (null/present) might result in significant changes in the function of the GST enzymes 1, 25, 27-33. The associations with these *GST* gene variants and cancer risk has been investigated in previous studies 34-36, particularly in association with the risk of developing glioma 11-13. However, the cumulative results remain inconclusive due to differences in ethnicity, the number of research subjects, patient age, and subtypes of glioma. There have been only two studies, but with inconsistent results, that have addressed the association with these *GST* gene variants and the risk of glioma in the Han Chinese population 11, 37. Therefore, the aim of this case-control study was to investigate the relationship between *GSTP1* (Ile105Val and Ala114Val), *GSTT1* (null/present) and *GSTM1* (null/present) variants and the risk of glioma in the Han Chinese population compared with the Caucasian population.

Due to the inconsistent findings in previously published studies regarding the association with *GST* gene variants and the risk of glioma, it was deemed necessary to perform a meta-analysis. At this time, there have been several previously published and formally analyzed systematic reviews and meta-analysis of published studies on the *GSTP1*, *GSTM1* and *GSTT1* variants and the risk of primary brain tumors, including glioma 1, 2, 38-40. However, the results of these previous meta-analyses are inconclusive, or have not been updated, or do not include racial subgroup analyses. Therefore, a meta-analysis was performed to specifically investigate the association with *GSTP1* (Ile105Val and Ala114Val), *GSTT1* (null/present) and *GSTM1* (null/present) variants and the risk of glioma that included data from this case-control study of 724 patients combined with other recently published and relevant studies.

## Materials and methods

### Case-control study subjects

The study subjects, including glioma patients and healthy controls, were enrolled from the Xiangya Hospital of Central South University (Changsha, China) between 2006 and 2013. All cases included in this study were histologically confirmed as glioma. The healthy controls were Han Chinese people, with no tumor and normal on physical examination and who were randomly selected from the Medical Examination Center of Xiangya Hospital.

The Chinese Clinical Trial Register approved the clinical study admission (Registration No. ChiCTR-COC-15006186), and the Ethics Committees of the Xiangya School of Medicine and the Institute of Clinical Pharmacology of Central South University approved the study protocol. A standardized questionnaire was used to collect demographic details and clinical data, including patient age, sex, smoking status, and glioma types. All the patients provided written informed consent in compliance with the Code of Ethics of the World Medical Association (Declaration of Helsinki) prior to participating in the study.

### Genotyping

DNA was isolated from 3ml of whole blood samples, using the phenol-chloroform extraction method, and then stored at 4°C. An amplification refractory mutation system assay, described by Hemmingsen *et al*, was performed to identify four alleles of *GSTP1* using two different sets of primers amplifying exon 5 and exon 6 41.

The primers included a forward primer upstream of the codon 105 substitution (5′-ACCCCAGGGCTCTATGGGAA-3′) and two reverse primers, primer 'A' (5′-TCACATAGTCATCCTTGCCGG-3′; Ala114-specific) and primer 'B' (5′-TCACATAGTCATCCTTGCCGA-3′; Val114-specific).

The polymerase chain reaction products were digested with the *Bsm*A1 restriction enzyme (New England BioLabs, Inc., Ipswich, MA, USA) and resolved using a 3.5% agarose gel to identify the Ile105Val variant. *GSTT1* and *GSTM1* null/present variants were detected by the polymerase chain reaction-restriction fragment length polymorphism (PCR-RFLP) method, as previously described 13.

### Meta-analysis literature and search strategy

Two investigators (MQZ and YJW) searched PubMed, PMC, EMBASE, Web of Science, and WanFang databases using the following search terms: 'glutathione S-transferase P1', 'glutathione S-transferase M1', 'glutathione S-transferase T1', 'GSTP1', 'GSTM1', 'GSTT1' combined with 'glioma' or 'brain tumor'. Studies that met the following inclusion criteria were included in the analysis: studies on *GSTP1*, *GSTM1*, *GSTT1* variants and risk of brain cancer, including glioma; and available genotypic frequencies. The exclusion criteria included: the lack of a control group; duplicate publications; and no available data on genotypic frequencies. Literature search updated on January 23, 2019. The information collected from each study included author name, year of publication, patient ethnicity, and frequency of variants in both groups. The Newcastle-Ottawa scale was used for quality assessment of literature reviewed 55.

### Statistical analysis

The SPSS software for Windows, version 13.0 (SPSS Inc., Chicago, IL, USA) was used for statistical analysis. The Hardy-Weinberg equilibrium was analyzed with the χ^2^ test. Data on age, sex and other characteristics were compared to glioma patients and healthy controls with the Student's t-test or χ^2^ analysis. The different distributions of the *GSTP1* variants of the two groups were examined by the χ^2^ test. P<0.05 was considered to indicate statistically significant differences. The meta-analysis was performed using STATA version 12 (Stata Corp, College Station, TX, USA).The significances of the pooled odds ratio (OR) were tested by the Z-test, and P<0.05 was considered to be statistically significant. Heterogeneity was calculated with the χ^2^ analysis based on the Q-test and I^2^. If P<0.05 and I^2^>50% were present, the Mantel-Haenszel random effect model was used, otherwise a fixed effect model was used 42, 43. Data was combined using fixed-effects models if the heterogeneity was significant. The potential publication bias was assessed using Egger's test and Begg's test.

## Results

### Patient characteristics

Of the 384 patients with glioma (194 men and 190 women) (mean age, 48.3 ± 9.2 years), and the 340 healthy controls (174 men and 166 women) (mean age, 46.5 ± 9.8 years) were enrolled in the case-controlled study. The demographic and clinical characteristics of the patients and controls are summarized in Table 1. The 384 patients included 128 (33.3%) cases of glioblastoma; 126 (32.8%) cases of aplastic astrocytoma, diffuse astrocytoma or other types of astrocytoma; and 130 (33.9%) cases of other gliomas. There was no difference in gender and age distribution between patient and controls.

### *GSTP1* (Ile105Val and Ala114Val), *GSTM1* (null/present) and *GSTT1* (null/present) variants and glioma risk

*GSTP1* (Ile105Val and Ala114Val), *GSTM1* (null/present) and *GSTT1* (null/present) variants were genotyped in 384 glioma patients and 340 healthy controls. The investigated SNPs were all in Hardy-Weinberg equilibrium. As shown in Table 2, the frequency of the Ile105Val GG genotype was significantly greater in the glioma group compared with that in the healthy controls (11.7% *vs.* 6.4%) (OR=1.50(1.05-2.04); P=0.01). The frequencies of the GA + AA genotypes were significantly different from the two groups (88.3 *vs.* 93.6%) (OR=1.47(1.04-2.10); P=0.015). There was no difference in the allele or genotype frequencies of Ala114Val (rs1138272) between glioma patients and healthy controls. As shown in Table 2, there were no differences in the frequencies of *GSTM1* (null/present) and *GSTT1* (null/present) variants between Han Chinese glioma patients and healthy controls: *GSTM1* (43% *vs.* 48%; OR=1.07(0.93-1.21); P=0.31); *GSTT1* (41.1% *vs.* 34.1%; OR=0.89(0.81-1.06); P=0.052).

### Meta-analysis study review and selection

**Figure 1:**
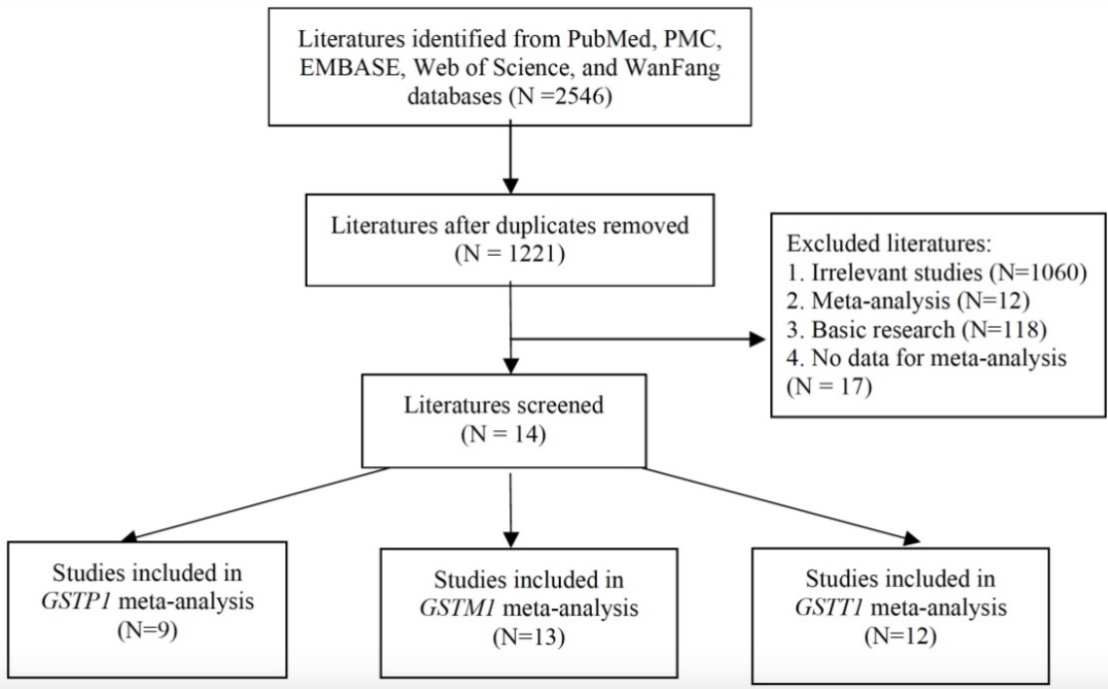
Selection of studies for inclusion in the meta-analysis.

Figure 1 shows the literature review selection procedure. A total of 2546 publications were selected from the PubMed, PMC, EMBASE, WanFang, and Web of Science databases. Following removal of duplicate publications and 1221 abstracts were retrieved. A total of 1060 irrelevant studies, 12 meta-analyses, 118 basic research studies, and e17 studies without detailed information were excluded, according to the publication selection criteria. Finally, 15 studies (including our case-control data) were deemed eligible for review (Table 3). There were 9 publications that included *GSTP1* variants, 13 publications that included *GSTM1* variants and 12 publications that included *GSTT1* variants. The characteristics of the included publications are summarized in Table 3.

### Meta-analysis about *GSTP1* Ile105Val and Ala114Val variants and glioma risk

**Figure 2:**
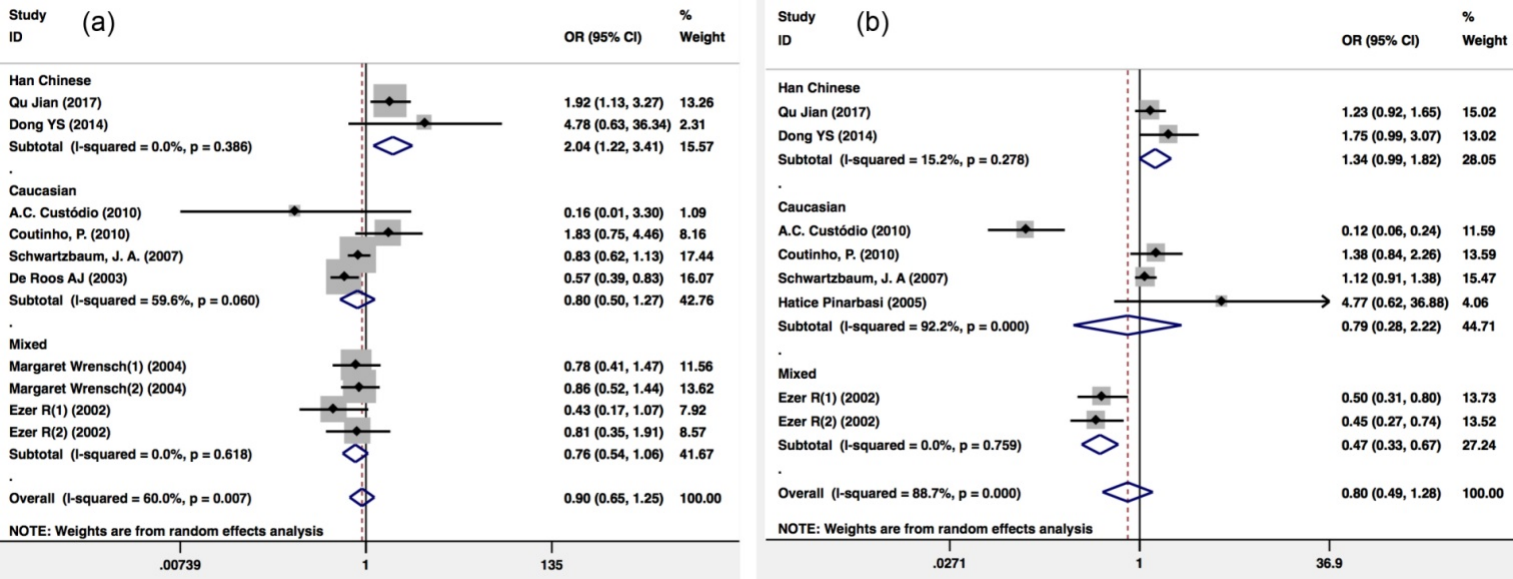
Meta-analysis of the *GSTP1* Ile105Val polymorphism and glioma risk. (a) Val/Val *vs.* Ile carriers; (b) Val carriers *vs.* Ile/Ile. CI, confidence interval.

A total of 9 publications cover *GSTP1* variants. According to the *GSTP1* Ile105Val recessive model (Val/Val *vs.* Ile carriers), the pooling ORs of eight publications including 4,128 glioma patients and 2,134 healthy controls showed a significant association. Because there was heterogeneity (I^2^=60%; P=0.007), a Mantel-Haenszel random-pooling model was used to analyze the published data. The results showed that there was significant difference risk of glioma between the *GSTP1* Ile105Val Val/Val genotype and Ile carriers among the Han Chinese population (OR=2.035(1.216-3.406); P=0.007) compared with that expected in the overall population (OR=0.902(0.650-1.251); P=0.535), the Caucasian population (OR=0.799(0.504-1.268); P=0.342) and a mixed population (OR=0.756(0.541-1.057); P=0.102) (Figure 2a).

There were 7 publications that included 3,265 glioma patients and 1,273 healthy controls that included the *GSTP1* Ile105Val variant dominant model (Val carriers *vs.* Ile/Ile). Because there was heterogeneity (I^2^=88.7%; P<0.001), a Mantel-Haenszel random-pooling model was used to analyze the published data. The results showed that the distribution of *GSTP1* Ile105Val Val variant carriers in glioma and healthy controls showed no difference (OR=0.797(0.495-1.285); P=0.352) (Figure 2b).

**Figure 3:**
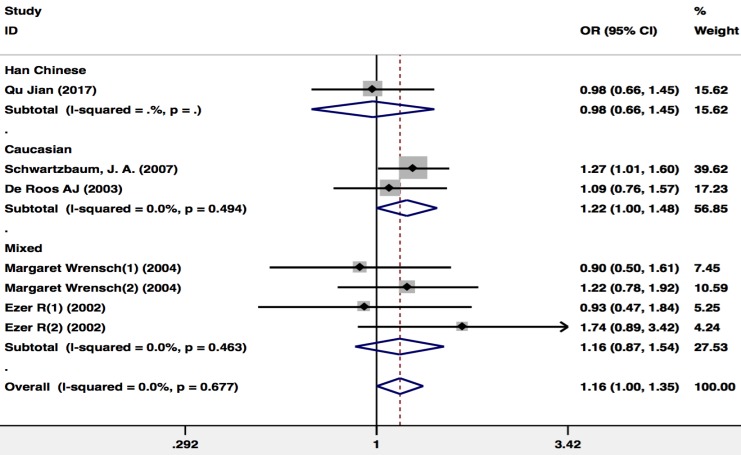
Meta-analysis of the *GSTP1* Ala114Val polymorphism and glioma risk (Val carriers *vs.* Ala/Ala).CI, confidence interval.

There were 5 publications including 2,342 glioma patients and 3,203 healthy controls included in the publications that included the *GSTP1* Ala114Val polymorphism dominant model (Val carriers *vs.* Ala/Ala). Because there was no study heterogeneity (I^2^=0%; P=0.677), a Mantel-Haenszel fixed pooling model was used for analysis. The* GSTP1* Ala114Val carriers were associated with an increased risk of glioma in the overall population (OR=1.163(1.002- 1.350); P=0.047) and in the Caucasian population (OR=1.215(1.001-1.476); P=0.049) (Figure 3).

### Meta-analysis about *GSTM1* (null/present) and *GSTT1* (null/present) variants and the risk of glioma

**Figure 4:**
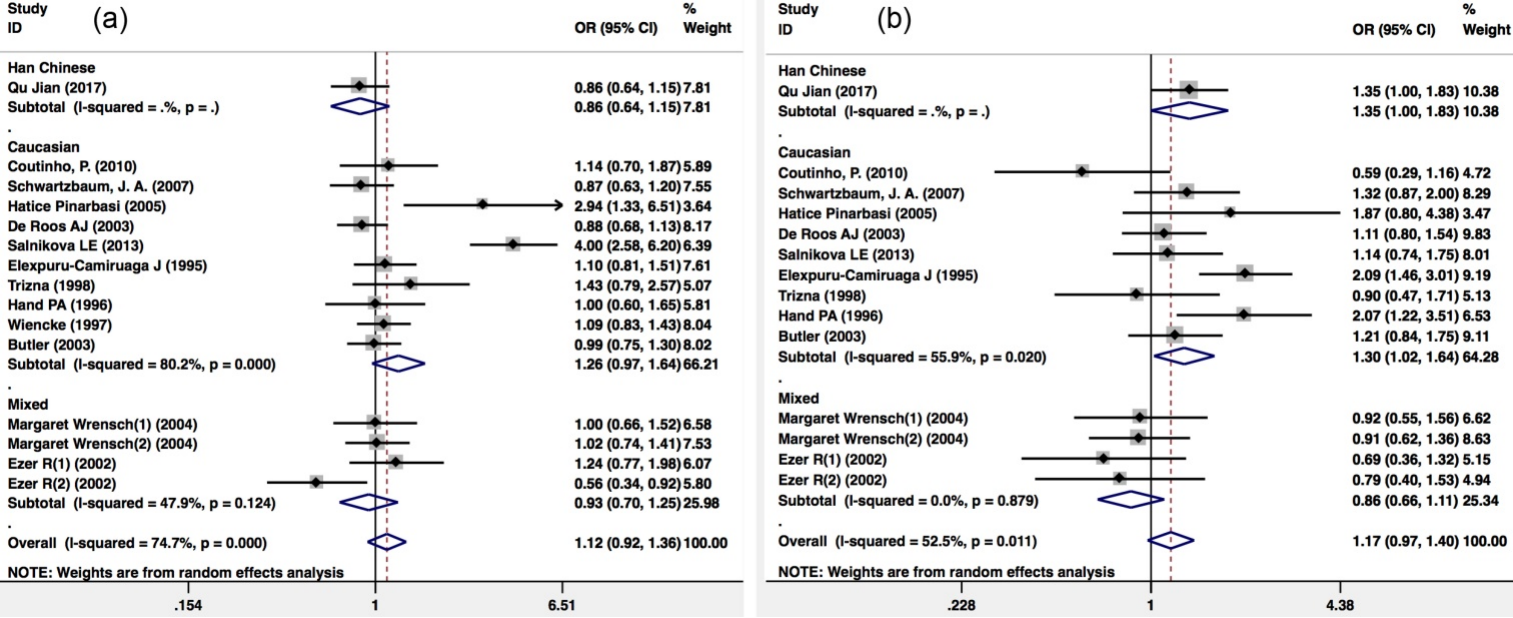
Meta-analysis of *GSTT1* and *GSTM1* null/present variants and glioma risk. (a) *GSTM1* null/present; (b) *GSTT1* null/present. CI, confidence interval.

There were 13 publications that included 2,312 glioma patients and 4,966 healthy controls included in the publication analysis of the *GSTM1* null/present variant and glioma risk. Because there was heterogeneity (I^2^=74.7%; P<0.001), a Mantel-Haenszel random-pooling model was used in the analysis. No significant association was found between the *GSTM1* null/present variant and glioma risk of the overall population (OR=1.121(0.924-1.360); P=0.247), the Caucasian population (OR=1.260(0.966-1.643); P=0.088) and the overall mixed population (OR=0.935(0.698-1.252); P=0.651) (Figure 4a).

There were 12 publications that included 2,830 glioma patients and 4,429 healthy controls included in the publication analysis that included the *GSTT1* (null/present) variant and glioma risk. Because there was heterogeneity (I^2^=52.5%; P=0.011), a Mantel-Haenszel random-pooling model was used in the analysis. There was no significant association with *GSTT1* (null/present) variant and glioma risk in the Caucasian population (OR=1.296(1.023-1.641); P=0.031) (Figure 4b).

### Publication bias and sensitivity analysis

Publication bias was examined by Egger's test and Begg's test. For all pooled OR analysis, Egger's test and Begg's test showed no publication bias (Figure S1). For the pooling ORs of *GSTP1* Ile105Val Val/Val *vs.* Ile carriers in the Han Chinese population and *GSTP1* Ala114Val Val carriers *vs.* Ala/Ala in the overall population and Caucasian population, sensitivity analysis showed that changing the analysis model (fixed or random model) had no significant effects on the pooled ORs and the final strength of the association (Table 4). For the pooling ORs of *GSTT1* null *vs.* present in Caucasians, sensitivity analysis found that changing the analysis model (fixed or random model) had no significant effect on the pooled OR and the final strength of the association.

## Discussion

The GST gene variants, *GSTP1*, *GSTT1,* and *GSTM1,* encode enzymes that metabolize carcinogenic compounds and reduced enzyme activity that may affect the risk of developing cerebral glioma. This case-control study investigated the associations with *GSTP1* (Ile105Val and Ala114Val), *GSTT1* (null/present) and *GSTM1* (null/present) variants and the risk of cerebral glioma. The study was supported by meta-analysis on *GST* gene variants and cerebral glioma in the Han Chinese population, which showed that *GSTP1* Ile105Val and *GSTT1* null/present variants were associated with the risk of glioma.

Susceptibility genes for the development of glioma have been identified with this study, and the findings are supported by previous studies. It has been previously reported that the rs25489 (Arg280His) and Arg399Gln (rs25487) polymorphisms of the *XRCC1* gene may affect the risk of developing glioma in the Chinese population 44. However, the development of cerebral glioma can only be partly explained by these gene findings, because glioma is a complex tumor, and its development may be associated with susceptibility to multiple genes as well as environmental factors.GST is known to catalyze the detoxification of reactive electrophilic compounds, such as cytotoxic drugs, and GSTs are a large family of cytosolic phase II xenobiotic metabolizing enzymes 45, 46. GST enzymes are associated with the accumulation of increased amounts of reactive oxygen species, which is a phenomenon involved in carcinogenesis 47. The individual variability in the GST enzyme activity may lead to different effects of detoxification of potential carcinogens and result in susceptibility to cancer 45, 46. The enzymes, GSTP1, GSTT1 and GSTM1 are the predominant enzymes in the GST enzyme family, and they have been proposed to play a critical role in protecting the brain from toxic compounds 48, 49. *GSTP1* I105V and A114V and deletions in the *GSTM1* and *GSTT1* genes could substantially reduce the activity of these protective GST enzymes 1, 32, 33, 35.

It has been previously reported that *GSTP1* is associated with susceptibility to other types of malignancy, including prostate cancer^50^ and breast cancer 31. Also, previous studies have shown that polymorphisms of the *GSTP1*gene may affect the response to chemotherapy and treatment outcome in breast cancer 51, and may also affect epirubicin treatment outcome as well as epirubicin-related toxicity 29. These findings indicate that the *GSTP1* gene is an important gene associated with the development and treatment of cancer.

Previous case-control studies assessed the association with *GSTP1* polymorphisms and the risk of developing glioma, but the reported findings have been inconsistent. Gao *et al.* analyzed the association with *GSTP1* polymorphisms and the risk of developing glioma in 301 patients with glioma and 302 healthy controls and reported a positive result for *GSTP1* Ile105Val (rs1695) 37. Casartelli*et al*. also found the *GSTP1* Ile105Val polymorphism to be involved in the susceptibility to astrocytoma and the development of glioblastoma multiforme (GBM) 12. However, there have also been studies reporting negative results 1, 2, 13. This inconsistency may be due to the differences in patient age, glioma subtype, and ethnicity*.* Some studies combined subtypes of glioma to analyze the distribution of *GSTP1* gene polymorphisms. Therefore, more patients are required to classify the subtypes of glioma and analyze the polymorphisms by subtype.

The results of this study were consistent with those of Qin *et al.* and Gao *et al.* both involving the Han Chinese population 11, 37. In the Qin *et al.* study, the distribution of rs1695 in *GSTP1* allelic frequencies was significantly different from 72 cases of GBM and 302 control cases in the Han Chinese population. Furthermore, rs1695 in *GSTP1* decreased the risk of GBM in the log-additive model (OR=0.56; 95% CI, 0.34-0.94; P=0.022) 11. In the present study, GBM comprised 33.3% of total glioma cases. Gao *et al*. investigated 301 glioma patients and 302 controls in a case-controlled study and found a positive association between *GSTP1* rs1695 and glioma risk. In the present study, Ile105Val GG genotype patients had a 1.5 times increase in risk compared with wild-type patients, and GG genotype patients had a 1.47 times increase in risk compared with AA+AG patients. Furthermore, both the literature review and the analysis undertaken in this study confirmed the positive associations.

Among the published genome-wide association studies (GWAS) relevant to the literature review and the analysis undertaken in this study, there was no direct information or data that could be applied to this analysis 52-57. A previous study reported that the* GSTP1* gene Ala114Val (rs1138272) polymorphism might be used as a predictive factor for cisplatin-based chemotherapy in osteosarcoma patients 30. Another study reported that children carrying *GSTP1* rs1138272 minor alleles might represent a susceptible population with increased risk of asthma associated with air pollution 58. These findings suggest that this variant may be involved in oxidative stress, which is a common phenomenon in the development of glioma. However, no association with this polymorphism with the risk of glioma was observed in the present study. However, meta-analysis undertaken in this study showed that *GSTP1* Ala114Val (rs1138272) was associated with the risk of glioma in the overall population and Caucasian population studied.

Ethnicity is an important factor associated with *GST* gene variants and glioma risk. In the meta-analysis in this study, a significant association was found between *GSTP1* Ile105Val (Val/Val *vs.* Ile carriers) and glioma risk in the Han Chinese population, but not in the Caucasian or mixed population. The *GSTT1* null/present variant was only significantly associated with increased risk of glioma in the Caucasian population. These results implied that the effects of *GST* gene variants affecting glioma was associated with ethnic differences. Further studies are required in future that includes populations of different ethnicities.

In the literature review and analysis undertaken in this study, there were several publications on that showed an association with *GSTP1* variants and the risk of glioma 1, 2, 38-40. The results for *GSTP1* Ile105Val (rs1695) were positive 37, but negative results were also reported 1, 2, 39, 40. The literature review and analysis undertaken in this study showed that the *GSTP1* Ala114Val variant was significantly associated with the risk of glioma 38, 39. The present study showed a positive result of *GSTP1* Ile105Val (rs1695). To draw more precise conclusions, meta-analysis was done to investigate *GST* gene variants and the risk of glioma.

Meta-analysis undertaken in this study included nine publications that showed no significant association with the *GSTP1* Ile105Val and Ala114Val variants. The funnel plots of the results of the three comparisons demonstrated that there was publication bias in the *GSTP1* Ile105Val analysis; this may have been due to the limited research samples and unpublished negative results. There were no robust results from the exact effects of the *GSTP1* variants. A widely expanded large-sample case-control study or GWAS may be necessary to identify the key pathogenic gene or genes associated with glioma.

Heterogeneity is an important element in the meta-analysis, and in this study, publication heterogeneity was found when the ORs were pooled for *GSTP1* Ile105Val (Val/Val *vs.* Ile carriers). To find the source of heterogeneity, an ethnicity subgroup analysis was performed and found significance only in the Han Chinese group of heterogeneity, which implied that ethnicity was part of the source of heterogeneity. Also, a random model and a fixed model were used to confirm the positive results. The Egger's test and Begg's test also showed no publication bias for all pooled OR analysis (Figure S1).

This study had several limitations. The study was performed in a single center and had a limited patient sample size. Because of the limited number of patients, the cases of glioma were combined, without distinguishing between the histological type of glioma, grade, stage, or isocitrate dehydrogenase (*IDH*) gene status. While there were some studies that found the different genetic susceptibility to glioma subgroups, such as GBM and non-GBM tumors, IDH-mutant and IDH wild-type tumors 59-61.

In conclusion, a case-controlled study, including a total of 724 subjects was performed, and showed that the *GSTP1* gene Ile105Val (rs1695) polymorphism was associated with an increased risk of glioma in the Han Chinese population, and the meta-analysis results confirmed this association and also showed that the *GSTP1* Ala114Val variant was associated with overall increased risk of glioma and an increased risk in the Caucasian population, and the *GSTT1* null/present variant was associated with an increased risk of glioma in the Caucasian population. Future large-scale, multi center, controlled, prospective studies are required to support these findings and to determine how these *GST* gene variants may affect the pathogenesis of cerebral glioma.

## Supplementary Material

Supplementary figures.

## Figures and Tables

**Table 1:** Demographic and clinical characteristics of the subjects.

Parameters	Patients, N (%) (N=384)	Healthy controls, N (%) (N=340)	P- value
Male gender	194 (50.5)	174 (51.2)	-
Female gender	190 (48.5)	166 (48.8)	0.84
Age (years), mean ± SD	48.3 ± 9.2	46.5 ± 9.8	0.73
Smoking (yes/no)	223/161	182/158	0.45
Glioma type			
Glioblastoma	128 (33.3)	-	-
Astrocytomas (except for glioblastoma)	126 (32.8)	-	-
Other	130 (33.9)	-	-

SD, standard deviation.

**Table 2:** Allele and genotype frequencies of the two SNPs in glioma patients (n=384) and healthy controls (n=340).

SNP	Genotype	Case	Control	P-value	Odds ratio
		(384)	(340)		(95%CI)
*GSTP1* Ile105Val rs1695	A	542(70.6%)	515(75.7%)		Reference
	G	226(29.4%)	191(24.3%)	0.31	1.06(0.94-1.20)
	AA	203(52.9%)	197(58.0%)		Reference
	AG	136(35.4%)	121(35.6%)	0.59	1.05(0.89-1.23)
	GG	45(11.7%)	22(6.4%)	0.01	1.50(1.05-2.04)
	AG+GG *vs.* AA	181(47.1%)	143(42%)	0.17	1.12(0.95-1.31)
	AA+AG *vs.* GG	339(88.3%)	318(93.6%)	0.015	1.47(1.04-2.10)
*GSTP1* Ala114Val rs1138272	C	687(89.5%)	613(90.1%)		Reference
	T	81(10.5%)	67(9.9%)	0.66	1.04(0.87-1.26)
	CC	322(83.9%)	284(83.5%)		Reference
	CT	43(11.2%)	45(13.2%)	0.45	0.92(0.74-1.14)
	TT	19(4.9%)	11(3.3%)	0.27	1.28(0.79-2.06)
	CT+TT *vs.* CC	62(16.1%)	56(16.5%)	0.91	0.99(0.80-1.22)
	CC+CT *vs.*TT	365(95.1%)	329(96.7%)	0.25	1.29(0.80-2.08)
*GSTM1*	Null	165(43%)	159(46.8%)	0.31	1.07(0.93-1.21)
	Present	219(57.0%)	181(53.2%)		Reference
*GSTT1*	Null	158(41.1%)	116(34.1%)	0.052	0.89(0.81-1.06)
	Present	226(58.9%)	224(65.9%)		Reference

Bold print indicates statistical significance; SNP, single-nucleotide polymorphism; CI, confidence interval.

**Table 3:** Characteristics of studies included in the meta-analysis.

Authors	Year	Ethnicity	NOS Score	*GSTP1* rs1695 Case			*GSTP1* rs1695 Control			*GSTP1* rs1138272 Case			*GSTP1* rs1138272 Control			*GSTM1* Control		*GSTM1* Case		*GSTT1* Control		*GSTT1* Case	
				AA	AG	GG	AA	AG	GG	CC	CT	TT	CC	CT	TT	(-)	(+)	(-)	(+)	(-)	(+)	(-)	(+)
Qu Jian	2017	Han Chinese	8	203	136	45	197	121	22	322	43	19	284	45	11	159	181	165	219	116	224	158	226
Dong YS^11^	2014	Han Chinese	7	180	102	19	52	19	1														
A.C. Custódio^12^	2010	Caucasian	7	87	13	0	35	43	2														
Coutinho, P.^13^	2010	Caucasian	6	129	172	46	35	37	6							153	194	37	41	76	271	11	67
Schwartzbaum, J. A.^14^	2007	Caucasian	9	682	716	193	216	189	67	580	138		1344	252		237	193	119	111	68	362	46	185
Hatice Pinarbasi^15^	2005	Caucasian	7	132	21		30	1								37	116	15	16	31	122	10	21
Margaret Wrensch(1)^16^	2004	Mixed	7	159		21	136		23	153	28		128	26		84	82	93	91	35	132	36	147
Margaret Wrensch(2)^16^	2004	Mixed	7	241		28	297		40	228	43		292	45		177	160	140	124	74	263	54	210
Ezer R(1)^17^	2002	Mixed	7	139	71	10	46	44	10	192	28	1	86	13	1	49	51	120	101	18	82	29	191
Ezer R(2)^17^	2002	Mixed	7	111	44	14	46	44	10	127	27	9	86	13	1	49	51	60	112	18	82	25	145
De Roos AJ^18^	2003	Caucasian	6	508		59	329		67	345	59		498	78		321	254	212	191	100	445	77	309
Salnikova LE^19^	2013	Caucasian	7													202	261	99	32	96	367	37	124
Elexpuru-Camiruaga J^20^	1995	Caucasian	6													351	262	130	88	91	403	70	148
Trizna^21^	1998	Caucasian	7													39	51	47	43	27	63	25	65
Hand PA^22^	1996	Caucasian	6													121	90	51	38	56	228	30	59
Wiencke^23^	1997	Caucasian	6													305	157	334	158				
Butler^62^	2003	Caucasian	8													294	285	164	161	85	494	56	269

**Table 4:** Meta-analysis of the association between *GSTP1*, *GSTM1* and *GSTT1* variants and glioma risk.

Genetic comparisons	No. of studies	Study groups	Test of association				Test of heterogeneity			
			OR/HR(95% CI)	Z	P-value	Model	χ2	P-value	I^2^(%)	Tau-squared
*GSTP1*										
Val/Val *vs.* Ile carriers	8	Overall	0.902(0.650-1.251)	0.62	0.535	R	22.5	0.007	60%	0.137
	2	Han Chinese	**2.035(1.216-3.406)**	2.7	**0.007**	R	0.75	0.386	0.00%	0
			**2.115(1.269-3.524)**	2.87	**0.004**	F				
	4	Caucasian	0.799(0.504-1.268)	0.95	0.342	R	7.42	0.06	59.60%	0.1085
	2	Mixed	0.756(0.541-1.057)	1.63	0.102	R	1.79	0.618	0.00%	0
Val carriers *vs.* Ile/Ile	7	Overall	0.797(0.495-1.285)	0.93	0.352	R	61.84	< 0.001	88.70%	0.3722
	2	Han Chinese	1.345(0.994-1.819)	1.92	0.055	R	1.18	0.278	15.20%	0.0095
	4	Caucasian	0.794(0.284-2.218)	0.44	0.66	R	38.38	< 0.001	92.20%	0.888
Val carriers *vs.* Ala/Ala	5	Overall	**1.163(1.002-1.350)**	1.98	**0.047**	F	4	0.677	0.00%	-
			**1.162(1.001-1.349)**	1.98	**0.048**	R				
	2	Caucasian	**1.215(1.001-1.476)**	1.97	**0.049**	F	0.47	0.494	0	-
			**1.216(1.002- 1.477)**	1.98	**0.048**	R				
	2	Mixed	1.160(0.871-1.543)	1.02	0.31	F	2.57	0.463	0	-
*GSTM1* null *vs.* present	13	Overall	1.121(0.924-1.360)	1.16	0.245	R	55.3	< 0.001	74.70%	0.102
	10	Caucasian	1.260(0.966-1.643)	1.71	0.088	R	45.52	< 0.001	80.20%	0.1372
	2	Mixed	0.935(0.698-1.252)	0.45	0.651	R	5.76	0.124	47.90%	0.0424
*GSTT1*null *vs.* present	12	Overall	1.168(0.974-1.400)	1.67	0.094	R	27.39	0.011	52.50%	0.0587
	9	Caucasian	**1.296(1.023-1.641)**	2.15	**0.031**	R	18.14	0.02	55.90%	0.0686
			**1.301(1.122-1.509)**	3.48	**0.001**	F				
	2	Mixed	0.857(0.660-1.112)	1.16	0.245	F	0.67	0.879	0.00%	-

OR, odds ratio; CI, confidence interval; vs., versus; F, fixed effect model; R, random effect model.
