# Supplementary material for: Glutathione S-transferase genes variants and glioma risk: A case-control and meta-analysis study
Source: J Cancer. 2019 Aug 19;10(19):4679–88. doi: 10.7150/jca.29398 (PMC6746118; doi:10.7150/jca.29398)

Glutathione S-transferase genes variants and glioma risk： A case-control and meta-analysis study

Weiping Liu[1, #], Hongyu Long[1], Mengqi Zhang[1], Yanjing Wang[2], Qiong Lu[3], Haiyan Yuan[3]， Qiang Qu[4], Jian Qu[3,#]

Additional supporting information

Figure S1 Begg's and Egger's bias plot for publication bias test. OR: odds ratio; SE: standard error. Begg's bias plot of (a) *GSTP1* Val/Val *vs.* Ile carriers, (b) *GSTP1* Val carriers *vs.* Ile/Ile and (c) *GSTP1* Val carriers *vs.* Ala/Ala; Egger's bias plot of (d) *GSTP1* Val/Val *vs.* Ile carriers, (e) *GSTP1* Val carriers *vs.* Ile/Ile and (f) *GSTP1* Val carriers *vs.* Ala/Ala.

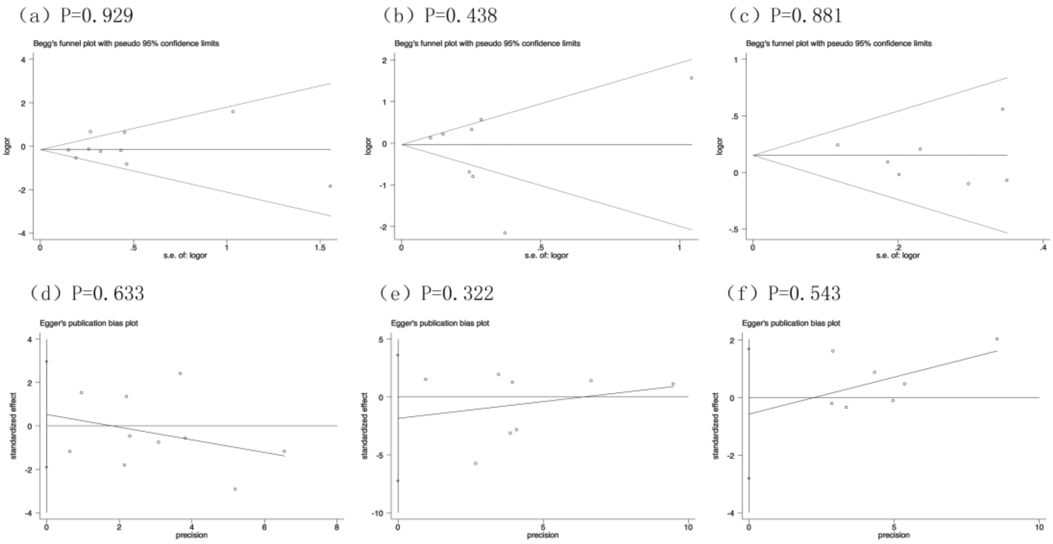

Figure S2 Begg's and Egger's bias plot for publication bias test. OR: odds ratio; SE: standard error. Begg's bias plot of (a) *GSTM1* null *vs.* present and (b) *GSTT1* null *vs.* present; Egger's bias plot of (c) *GSTM1* null *vs.* present and (d) *GSTT1* null *vs.* present.

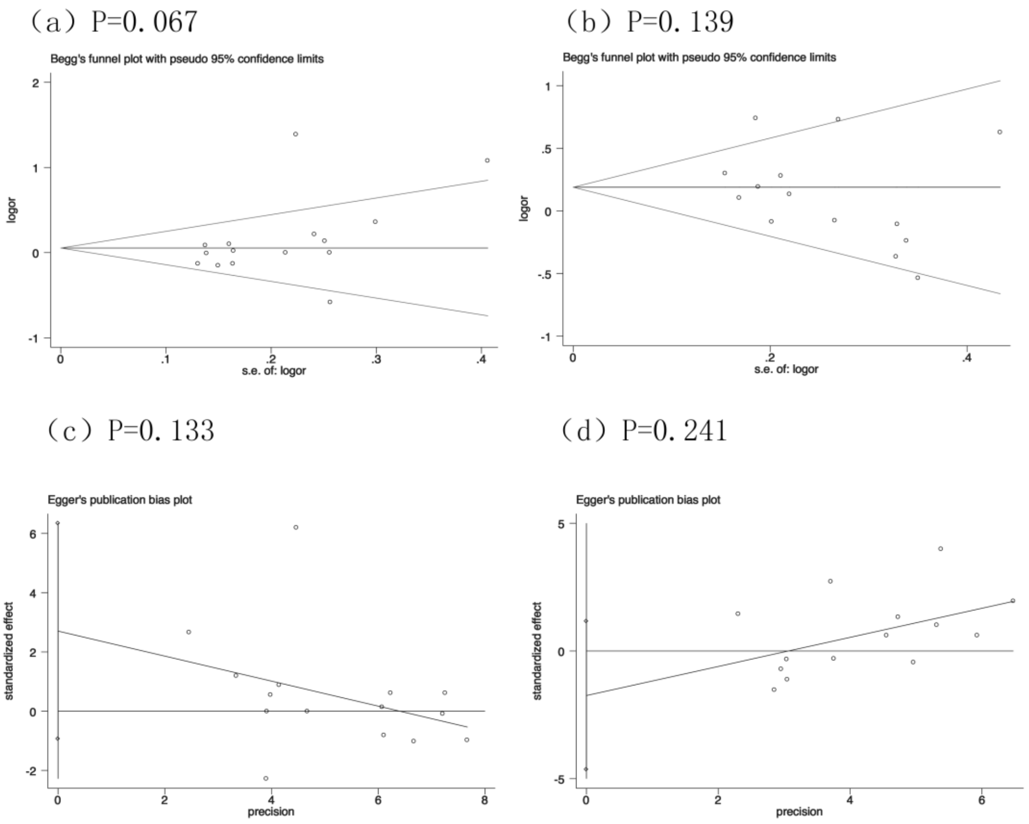

Supplement: Supplementary file 1 — Supplementary figures. [file jcav10p4679s1.pdf]
